# The Alteration in Composition and Function of Gut Microbiome in Patients with Type 2 Diabetes

**DOI:** 10.1155/2020/8842651

**Published:** 2020-11-11

**Authors:** Xue Zhao, Yiding Zhang, Ruixue Guo, Wei Yu, Fanliang Zhang, Feng Wu, Jin Shang

**Affiliations:** ^1^Department of Nephrology, The First Affiliated Hospital of Zhengzhou University, No. 1 of East Jianshe Road, Zhengzhou, Henan, China 450052; ^2^Department of Nephrology, Shandong Provincial Hospital, Cheeloo College of Medicine, Shandong University, Jingwuweiqi 324, Huaiyin District, Jinan, Shandong, China

## Abstract

**Background:**

Diabetes mellitus (DM) has become one of the most common chronic metabolic diseases worldwide. Due to the increasing prevalence and various complications, diabetes brings about a huge financial burden to DM patients. Nowadays, more and more studies reveal the relationship between diseases and gut microbial community. We aimed to explore the alteration in composition and function of the gut microbiome in T2DM patients.

**Methods:**

A total of 137 patients with diabetes and 179 age- and gender-matched healthy controls selected from the healthy people sample center in the First Affiliated Hospital of Zhengzhou University were divided into the DM group and the Con group, respectively. We collected their venous blood for laboratory tests and stool samples for 16S rRNA sequencing. The comparison between the two groups including both composition and function of the gut microbiome is presented.

**Results:**

We found that the *α*-diversity of bacterial taxa in the DM group had an evident decrease compared to that in the Con group. At the phylum level, the DM group had an obvious decrease of *Bacteroidetes* and a marked increase of *Proteobacteria*, *Actinobacteria*, *and Verrucomicrobia*. At the genus level, *Bacteroides* and *Prevotella* decreased the most while *Escherichia-Shigella*, *Lachnospiraceae_incertae_sedis*, *Subdoligranulum*, *Enterococcus*, and *Klebsiella* had different degrees of expansion in the DM group. The ROC based on 246 optimum OTUs had very high test efficiency with an AUC of 92.25% in the training set and 90.48% in the test set. As for prediction of metabolic function, the gut microbiome of DM patients was predicted to be more active in environmental information processing and human diseases but less in metabolism.

**Conclusion:**

We observed alteration of composition and function of the gut microbiome in the DM group. These changes may provide a new treatment strategy for DM patients and new research targets.

## 1. Introduction

Diabetes mellitus (DM) is one of the most common metabolic diseases worldwide, characterized by hyperglycemia. Because of its rising prevalence, DM has become a worldwide health problem which brings tremendous financial burden. The International Diabetes Federation declares that diabetes will affect 642 million people worldwide until 2040. Millions of patients with DM have various complications such as retinopathy, kidney disease, and neuropathy, causing substantial pain to the patients.

Gut microbiota involvement is increasingly recognized to be associated with various diseases in recent years. These bacteria parasitically living in the human gut may indicate that the host is in a state of disease when the composition and proportion of the gut flora changed. Some studies find the relationship between DM and gut microbiome composition [1]. What is more, the gut-kidney axis is a new research direction nowadays, which may indicate the relationship between the gut microbiome and renal diseases including diabetic nephropathy [2].

According to recent studies, the relationship between DM and gut microbiome has attracted more and more attention. The pharmacodynamic effects of *Sophora flavescens* EtOAc extract on high-fat diet and low-dose streptozotocin-induced T2DM rats were examined, and the results showed that the *Sophora flavescens* EtOAc extract may have an effect on T2DM by mediating the host-microbial metabolic axis [3]. Several reported microbial metabolites related to the gut microbiota were associated with an increased risk of incident type 2 diabetes, reducing insulin secretion and insulin sensitivity [4].

Nevertheless, the certain relationship between gut microbiota involvement and diabetes is still not clear. The alteration of the bacterial community in DM patients is not completely studied either. How the diversity of the gut microbiome plays a role in the process of diabetes mellitus is also waiting to be explored. The aim of our project is to find the difference in the gut microbiome between DM patients and healthy controls, both in terms of composition and function. By means of analysis of the gut flora, our purpose is to provide new effective targets of diagnosis or treatments for diabetes.

## 2. Design and Methods

### 2.1. Ethical Approval

All subjects included in this project provided written informed consents. The First Affiliated Hospital of Zhengzhou University Ethics Review Committee granted ethical approval for the study.

### 2.2. Participant Identification and Sample Collection

In this population-based cross-sectional study, a total of 137 patients (18-75 years old) with DM were enrolled, who were admitted in the First Affiliated Hospital of Zhengzhou University from October 2018 to October 2019. The diagnostic criteria of diabetes mellitus were as follows: (1) twice fasting plasma glucose (FPG) ≥ 7.0mmol/L, (2) twice oral glucose tolerance test (OGTT ≥ 11.1mmol/L), and (3) diabetic symptoms (polyuria, thirst, drinking more water, and unexplained weight loss) accompanied with twice random blood glucose ≥ 11.1mmol/L. The DM group consisted of all enrolled DM patients for the following research.

Fecal samples of 179 age- and gender-matched healthy controls were obtained at the healthy people sample center of the First Affiliated Hospital of Zhengzhou University. The Con group consisted of these healthy volunteers for comparison with the DM group. The exclusion criteria were as follows: (1) long-term antibiotic application, (2) disease of the digestive system, and (3) recent history of infection.

Besides fecal samples, we also obtained clinical data including their basic information (age, gender, SBP, and DBP) and laboratory examination (eGFR, Hb, GHb, Cr, ALB, 24 h-pro, GRAN, LYM, and T/Cr) from our public clinical database.

The fecal samples and venous blood samples of all participants were collected after 12 h of overnight fasting. After being collected, all samples were temporarily stored in ice packs and immediately brought to the laboratory to be frozen at a -80°C environment within 2 hours for further analysis.

### 2.3. DNA Extraction, 16S rRNA Amplification, and Gene Sequencing

DNA extraction was completed using the E.Z.N.A.® Stool DNA Kit. The process of DNA extraction was referred to a previous study of Peng et al. [5].

The 16S rRNA had highly conservative V3-V4 region, which was amplified by PCR so that we got purified amplicons for MiSeq sequencing. All procedures were completed by Shanghai Mobio Biomedical Technology Co. Ltd. using the MiSeq platform (Illumina Inc., USA) according to the manufacturer's protocols. In the end, we compared our data with the 16S Bacteria and Archaea Ribosome Database to determine the changes.

### 2.4. Annotation of OTU

After chimeric sequences were removed using UPARSE (version 7.1, http://drive5.com/uparse/), we classified operational taxonomic units (OTUs) based on 97% similarity. To analyze the phylogenetic affiliation of 16S rRNA gene sequence, we used RDP Classifier (http://rdp.cme.msu.edu/) against the Silva (SSU123) 16S rRNA database with confidence threshold of 70%.

### 2.5. Bioinformatic Analysis

The Shannon rarefaction curve was used to estimate whether OTU richness basically approached saturation in all samples and compare the microbial diversity in the DM and Con groups. We used the nonparametric Shannon-Wiener (SW) diversity index and Simpson diversity index to assess sample diversity metrics. The nonparametric Mann–Whitney *U* test was used to compare OTU differences of the two groups. The Venn diagram was used to compare the differences in intestinal flora based on OTUs between the two groups. A nonparametric Kruskal-Wallis test was used as well. Both weighted and unweighted UniFrac were calculated in QIIME. In addition, principal coordinate analysis (PCoA) plots were also generated by the QIIME pipeline to visualize the unweighted UniFrac dissimilarity. Permutational multivariate analysis of variance (PERMANOVA) was used to check the statistical significance difference of the two groups by using 10,000 permutations. We subsequently used the linear discriminant analysis (LDA) effect size (LEfSe) to detect differential abundance at the taxon level. Taxa were shown if LDAvalues > 2.0 with a *P* value < 0.05. Phylogenetic Investigation of Communities by Reconstruction of Unobserved States (PICRUSt) was used to identify significantly different metabolic pathways and orthologous groups between the DM and Con groups compared with the KEGG database (LDAscores > 2.0 with a *P* value < 0.05).

### 2.6. Gut Microorganism-Based ROC Construction

In order to make sure whether the gut flora can be used to distinguish diabetic patients from healthy people, the participants were randomly divided into the test group (DM = 102, Con = 131) and the training group (DM = 35, Con = 48). We used LEfSe to evaluate all OTUs to obtain optimum OTUs with an LDAscore > 2.0. Based on the differences of obtained OTUs in the two groups, we tried to establish the operating characteristic curve (ROC) to verify the diagnosis efficacy of gut microbiota in DM patients and healthy controls. The area under ROC curve (AUC) values were generated in R (http://www.R-project.org/).

## 3. Results

In our project, we collected fecal samples from 316 volunteers consisted of 137 patients with DM and 179 healthy controls. According to their different bioinformatic analysis and clinical diagnosis, a total of 316 fecal samples enrolled were divided into the DM (*n* = 137) or Con (*n* = 179) group. The microbial community between DM and Con was compared to find out the difference in the two groups. In terms of metabolism, we identify different KEGG (Kyoto Encyclopedia of Genes and Genomes) pathways and orthologous groups (Ko) between DN and MN patients by means of PICRUSt (Phylogenetic Investigation of Communities by Reconstruction of Unobserved States).

### 3.1. Baseline Characteristics of Participants

The results of laboratory examination are shown in Table 1. SBP, DBP, GHb, GRAN, 24 h-pro, and T/Cr were observed to be markedly increased in the DM group (*P* < 0.05), while Hb and ALB had an obvious decrease in the DM group (*P* < 0.05). However, there was no significantly statistical difference in eGFR, Cr, and LYM between the two groups (*P* > 0.05).

### 3.2. Microbial Diversity of the DM or Con Group

**Figure 1:**
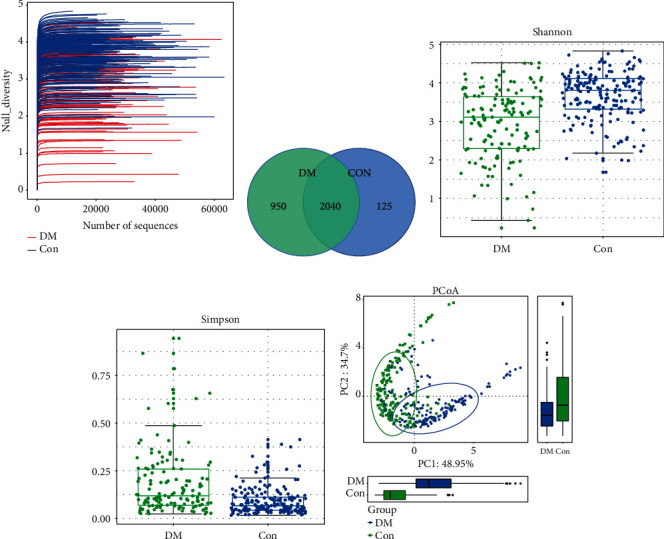
Comparison of microbial diversity and species composition between DM (*n* = 137) and Con (*n* = 179). (a) Shannon-Wiener curves showing estimated OTU richness basically approached saturation in all samples, and the microbial diversity was lower in the DM group. (b) Venn diagrams showing OTU distribution in different groups. (c, d) Estimated by the Shannon and Simpson index *α*-diversity in the DM or Con group. (e) Based on weighted UniFrac algorithm, *β*-diversity was visualized among the two groups in way of PCoA. DM: diabetes mellitus; Con: control group; OTUs: operational taxonomic units; PCoA: principal coordinate analysis.

**Figure 2:**
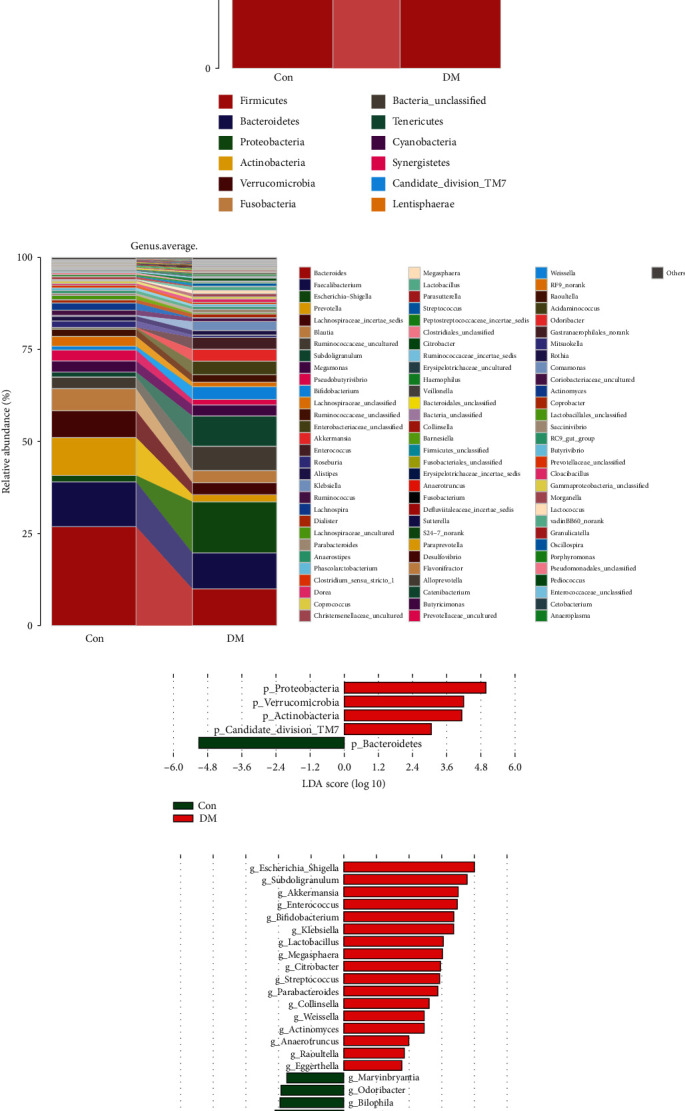
Composition of the gut microbiome in the DM or Con group. (a, b) Distribution of differential flora at the phylum and genus levels between the DM and Con groups. (c, d) LEfSe analysis of microbial profiles between DM and Con at the phylum and genus levels. MN, DM: diabetes mellitus; Con: control group; OTUs: operational taxonomic units; LDA and LEfSe: linear discriminate analysis and effect size.

We analyzed the microbial diversity of the DM and Con groups. The Shannon-Wiener curve based on OTUs had already been flat, indicating that our sequencing depth had already been adequate (Figure 1(a)). The similarity and overlap of bacterial community composition in the two groups were shown in the Venn diagram (Figure 2(b)). Using the Shannon and Simpson index, we found that the *α*-diversity of bacterial taxa in the DM group had a decrease compared to that in the Con group (Figures 1(c) and 1(d)). After calculating by weighted UniFrac algorithm, the *β*-diversity of the two groups is shown in Figure 1(e) by means of PCoA. The result indicated that the components of PC1 and PC2 could account for 83.65% proportion of total variance in two dimensions.

### 3.3. The Differences in Composition of Gut Microbiome in the DM or Con Group

The average relative abundance of the gut microbiome at the phylum and genus levels in the two groups is shown in Figures 2(a) and 2(b).

At the phylum level, there was an obvious decrease of *Bacteroidetes* in the DM group compared to the Con group. However, *Proteobacteria* and *Actinobacteria* had an evident increase in the DM group (Figure 2(a)).

In terms of the genus level, *Bacteroides* was found to have a considerable decrease in DM, followed by *Prevotella* and *Lachnospiraceae_incertae_sedis*. Nevertheless, compared to the Con group, *Escherichia-Shigella*, *Subdoligranulum*, *Akkermansia*, and *Enterococcus* were found enriched in the DM group (Figure 2(b)).

By means of LEfSe analysis, the gut microbiome with significant differences in richness at the phylum and genus levels is shown in Figures 2(c) and 2(d). At the genus level, a total of 36 genes of bacteria had a significant difference with an LDA score more than 2.0 and even 15 of them had an LDA score more than 3.6.

### 3.4. Area under the ROC Curve (AUC) Values

**Figure 3:**
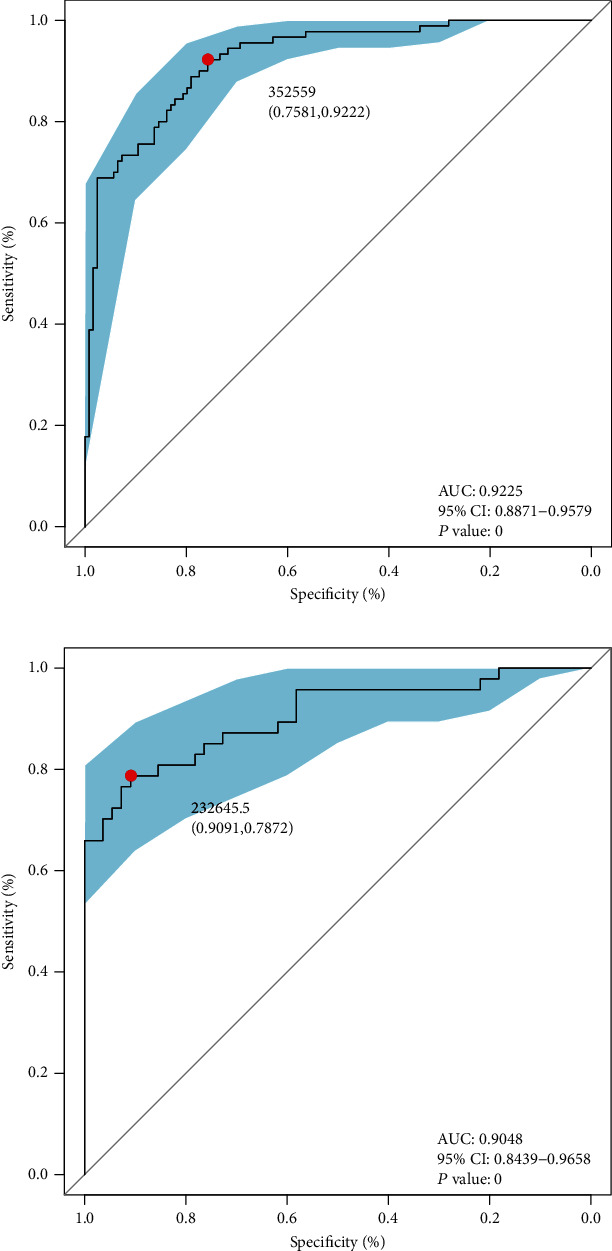
Differential capability based on obtained OTUs. (a) ROC curves based on obtained microbial markers showing discrimination rate for DM and Con in the training set. AUC, 95% CI, and *P* value were listed in the graph. (b) ROC curves based on obtained microbial markers showing discrimination rate for DM and Con in the test set. AUC, 95% CI, and *P* value were listed in the graph. ROC: receiver operating characteristic curve; AUC: area under the curve; CI: confidence intervals.

By means of LEfSe analysis, we evaluate a total of 3115 OTUs to obtain optimum OTUs with an LDAscore > 2.0. As a result, we obtained 246 OTUs which were different in abundance between the two groups with an LDAscore > 2.0. Based on these differential OTUs, we established the ROC to evaluate whether there was any difference in the gut microbiome between DM patients and healthy individuals. Surprisingly, we found that these 246 optimum OTUs had very high test efficiency with an area under the ROC curve (AUC) of 92.25% (95% CI: 88.71% to 95.79%; cut-off value: 352559, sensitivity: 0.9222, specificity: 0.7581; Figure 3(a)) in the training set. We subsequently verified after the same method in the test set, and the result showed the similar efficiency with an area under the ROC curve (AUC) of 90.48% (95% CI: 84.39% to 96.58%; cut-off value: 232645.5, sensitivity: 0.7872, specificity: 0.9091; Figure 3(b)).

### 3.5. Functional Alteration of Gut Microbiome in the DM and Con Groups

**Figure 4:**
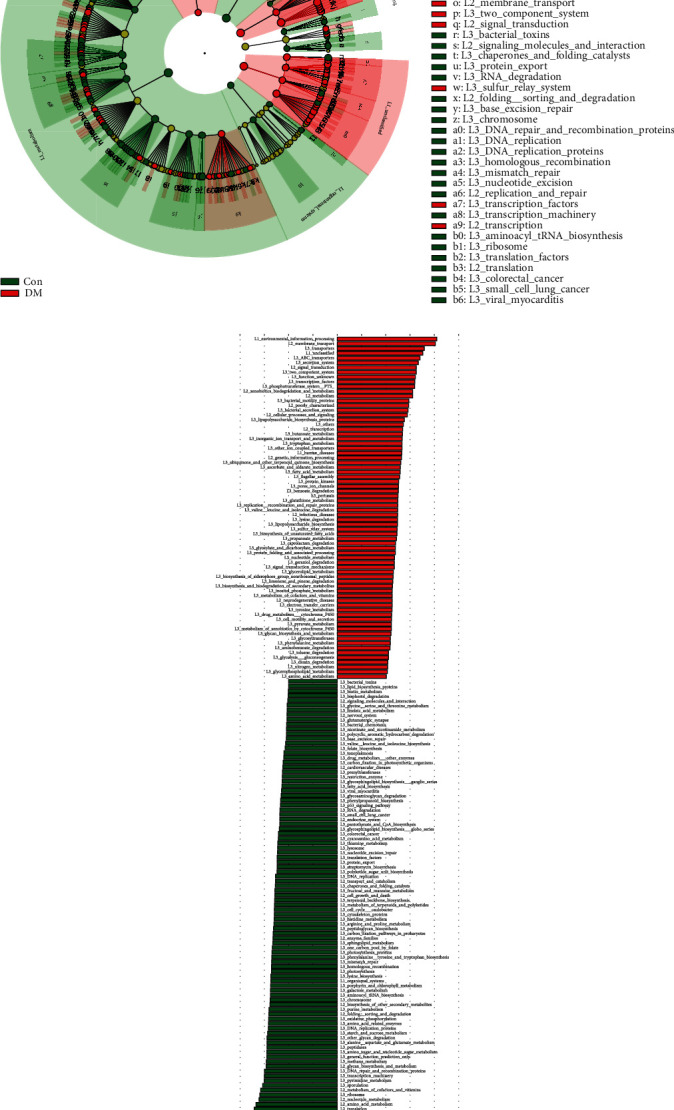
Predicted KEGG pathways. (a) LEfSe analysis showing different metabolic pathways between DM and Con. (b) LEfSe analysis showing different metabolic enzymes between DM and Con. KEGG: Kyoto Encyclopedia of Genes and Genomes; LEfSe: linear discriminate analysis and effect size.

PICRUSt was used to identify different KEGG pathways and orthologous groups (Ko) between the DM and Con groups (Figures 4(a) and 4(b)). Compared to the Con group, the functional alteration of the gut microbiome in the DM group was enormous. Among the six major metabolic pathways, environmental information processing and human diseases were observed to be significantly increased in the DM group (LDA > 2.0). Nevertheless, metabolism, genetic information processing, and organismal systems were predicted to be more active in the Con group (LDA > 2.0). For example, it was more active in membrane transport and signal transduction in the DM group while the translation and energy metabolism were decreased. At the same time, the DM group had more alteration in environmental information processing such as xenobiotic biodegradation and metabolism.

## 4. Discussion

In the current study, we analyzed the 16S rRNA sequencing data of fecal microbiome from a large cohort of individuals composed of DM patients and healthy controls. The results indicated that it was dysbiosis of gut microbial profile in the DM group compared to the Con group. Then, we analyzed the difference and similarity of the gut microbiome in the two groups and acquired the differential bacteria at the phylum and genus levels. The function alteration of microbiome was also researched in a way of KEGG, which might provide a new target in delaying or treating diabetes mellitus. In addition to the composition of the gut flora, we also explored the relationship between the gut microbiome and results of laboratory examination, aiming to provide direction for clinical treatment. What is more, the exploration about the function of bacterial genome was another innovation of our study, trying to offer new ideas to future research.

By means of 16S rRNA gene sequencing, the diversity analysis of the two groups revealed that the diversity of the DM group significantly decreased compared to the Con group, which was consistent with other research [6]. The explanation for this phenomenon might be that the patients with diabetes had intestinal microflora disorder and the application of metformin, which might aggravate the dysbiosis of gut microbial profile in DM patients.

At the phylum level, we found that *Bacteroidetes* was obviously decreased in the DM group, which was in accordance with the study of Wang et al. [7]. Nevertheless, *Proteobacteria*, *Verrucomicrobia*, and *Actinobacteria* were found increased in varying degrees. *Firmicutes* were found to have no significant differences between the two groups. Studies showed that obesity and dyslipidemia were related to the low prevalence of phylum *Bacteroidetes* and the increase of *Firmicutes/Bacteroidetes* ratio [8], which may be a contributor to changes of blood lipid levels in diabetic patients.

What is more, we found more alterations of microbiome abundance at the genus level including the decrease of *Bacteroides*, *Blautia*, *Faecalibacterium*, *Lachnospira*, *Pseudobutyrivibrio*, and *Roseburia* and the increase of *Escherichia-Shigella*, *Subdoligranulum*, *Akkermansia*, *Enterococcus*, *Bifidobacterium*, *Klebsiella*, *Lactobacillus*, and *Megasphaera* by means of LEfSe with an LDAscore > 3.6.

The decrease of phylum *Bacteroidetes* was mainly due to the poor abundance of *Bacteroides* in the DM group at the genus level. *Bacteroides* was famous for its genomic capability, which could ferment various sugars into short-chain fatty acids (SCFAs) [9]. Among SCFAs, propionic acid could reduce visceral fat. Both acetic acid and propionic acid were associated with the mechanisms related to maintaining or changing glucose homeostasis [10]. Therefore, we surmised that the decrease of *Bacteroides* was one of the important reasons for causing glucose homeostasis disorder in DM patients.

*Lachnospiraceae* was a kind of anaerobic, spore-forming bacteria, which was one of the most abundant microbiome in the human gut. After helping the host to digest some carbohydrates and fibers, some members of this genus could produce butyric acid in the human colon. It was reported that in animal models with type 2 diabetes, butyric acid played a key role in inducing GLP-1 production in the intestine and protect intestinal barrier function to improve glucose homeostasis in the host [11]. What is more, benefiting from its capability to inhibit some certain proteins to produce inflammation, butyric acid had a great effect on promoting anti-inflammation. Particularly, it helped to control inflammatory immune response by regulating T cells which could promote inflammation [12].

Similar to *Bacteroides*, *Blautia*, *Faecalibacterium*, *Pseudobutyrivibrio*, and *Roseburia*, bacterium-producing short-chain fatty acids (SCFAs) were all less abundant in the DM group. In a multicenter, randomized, open-label clinical trial, metformin and the Chinese herbal formula significantly increased a coabundant group represented by *Blautia* spp., which significantly correlated with the improvements in glucose and lipid homeostasis [13].

The average relative abundance of *Prevotella* was found significantly decreased in the DM group, which seemed to be inconsistent with Leite et al.'s study [14]. Nevertheless, *Prevotella* was found to have no statistical difference in the cladogram analyzed by LEfSe in our study. It might be because that the relative abundance of *Prevotella* in several samples from DM patients was at an extremely low level. *Prevotella* also belonged to phylum *Bacteroidetes*. The reason for these differences might be the different diet. Studies had demonstrated that the abundance of *Prevotella* in feces was related to high fiber and high carbohydrate dietary intake [15]. High-fiber diet could improve glucose homeostasis and increase *Prevotella* levels in feces [16]. *Prevotella* had great power to ferment dietary polysaccharides [17]. It also played a key role in intestinal inflammation [18]. Studies demonstrated that *Prevotella* predominantly activated TLR2 receptors and induced Th17 CD4 T cell polarization. Moreover, *Prevotella* also had an effect on inducing IL­8 and IL­6 secretion by epithelial cells, favoring Th17 responses and neutrophil recruitment [19]. Benefiting from its various functions, *Prevotella* was supposed to be an attractive candidate for the development of probiotic-based therapies in diabetes [20].

There were also several abundant genera (*Escherichia-Shigella*, *Subdoligranulum*, *Akkermansia*, *Enterococcus*, *Bifidobacterium*, *Klebsiella*, *Lactobacillus*, and *Megasphaera*) enriched in the DM group. *Escherichia-Shigella* was found to increase the most in fecal samples of the DM group, which was consistent with a previous study [21]. *Escherichia-Shigella* was considered to be a kind of opportunistic pathogen for humans, which could produce various proinflammatory components such as lipopolysaccharide (LPS) and peptidoglycans (PGN) and finally trigger host immune response and lead to intestinal inflammation in varying degrees [22]. What is more, as proinflammatory bacteria, *Escherichia-Shigella* exposed subjects to the impaired epithelial integrity, leading to low-grade inflammation and autoimmune responses, which might increase the risk of type 1 diabetes [23].

*Subdoligranulum* are anaerobic, spore-free Gram-negative bacteria. In the research about other diseases, *Subdoligranulum* was found to be tightly associated with chronic inflammation-related immune markers [24].

Within the past decade, intestinal symbiotic bacterium *Akkermansia muciniphila*, a species of *Akkermansia*, had emerged as a “sentinel of the gut,” which was considered as a sensitive indicator of intestinal permeability [25]. It had great effect on improving glucose tolerance [26] and could help in obesity to alleviate fat inflammation [27]. However, inconsistent with previous observations, *Akkermansia* was found obviously increased in the DM group. The reason for the abnormal increase of *Akkermansia* might be due to the application of metformin in diabetic patients. Studies had shown that the application of metformin could increase the abundance of *Akkermansia* in a mouse model [28]. However, diet might be another explanation of this phenomenon. Gurley et al. reported that after providing with green tea whose levels of polyphenols were comparable to those consumed by humans, the abundance of *Akkermansia muciniphila* was observed significantly increased [29]. Whether the abnormal increase we observed could be attributed to the effect of metformin application and different diet remained to be verified.

*Enterococcus* and *Klebsiella* were the conditioned pathogens that existed in the human gut. A previous study found that *Enterococcus* was positively correlated with obesity [30], and *Enterococcus* tended to cause urinary tract infection when the host had a weakened immune status. *Klebsiella* was considered to be associated with lung infection clinically. We surmised that due to the intestinal microflora disorder in diabetic patients, both of them snatched more living space and nutrients. Thus, they were found to be more abundant in the DM group in our study.

*Bifidobacterium* and *Lactobacillus* genera were generally recognized as beneficial bacterium. In a recent research, dietary pistachio restored normal flora and enhanced the presence of beneficial microbes including *Bifidobacterium* and *Lactobacillus* genera in the rat model of streptozotocin-induced diabetes [31]. In obesity rats, both *Bifidobacterium* and *Lactobacillus* were found to have an obvious decrease [32]. Hussain and his colleague found that *Lactobacillus plantarum LB818*, a species of *Lactobacillus*, could effectively ameliorate body weight gain and decrease total body fat by regulating fasting glucose levels in HFD-fed mice by reducing alanine aminotransferase (ALT), total cholesterol (TC), and triglyceride (TG) and elevating high-density lipoprotein levels in serum and decreased deposition of fat droplets in the liver [33].

*Megasphaera* was also found increased in patients with gestational diabetes mellitus [34]. In a study of Gaike et al., it was reported that the abundance of *Megasphaera* was increased in DM patients with long-term use of medication positively associated with fasting glucose and HbA1c [35]. We inferred that it might be owed to the function of fermentation fructose and lactic acid of *Megasphaera*.

Based on those discriminatory microbial OTUs, we established the ROC with high AUC (92.25%) in the training set to evaluate the microbial differences in the DM group and the Con group. What is more, we verified the result in the test group and successfully obtained the similar result with an AUC of 90.48%. The high test efficiency of ROC could not only be used to prove the microbial differences in the two groups but also to distinguish DM patients from healthy people.

As for prediction of the metabolic function of bacteria, the gut microbiome in the DM group was predicted to be more active in environmental information processing and human diseases. We inferred that the increase of environmental information processing was associated with the increase of membrane transport. Along with the alterations of composition of intestinal microbes, the environment of gut such as pH value and humidity was also changed. Thus, intestinal microbes would be more active in environmental information processing. In terms of the increase of human diseases, it might be mainly because of the growth of opportunistic pathogens such as *Escherichia-Shigella* and *Klebsiella*, which would increase the risk of host disease. However, metabolism was observed decreased in the DM group. It might be related to the decrease of production and metabolism of short-chain fatty acids mentioned previously. The exploration about alteration in metabolic function of the gut microbiome might provide new ideas for treating type 2 diabetes mellitus.

Our study had a number of advantages. First of all, our project had the largest sample size as far as we know, reducing the influence of individual error on the results. What is more, we combined gut microbiome with laboratory examination and discussed the influence due to the change of flora composition on diabetic patients. We also tried to explore the functional change of the gut microbiome so that we hoped to provide new treatment target for type 2 diabetes mellitus.

On the other hand, there were also several limitations. We did not have multicenter external verification to reduce the race and diet influence on outcomes. Owing to the lack of animal and cytological experiments, our study was unable to explain the mechanism of the alteration and effects of the gut microbiome.

## 5. Conclusion

All in all, the fecal microbial community had an obvious alteration in patients with T2DM. Our research found the alteration of the gut microbiome both in composition and function in patients with type 2 diabetes mellitus. The result of ROC fully proved our result and could even be used to help distinguish diabetics from healthy individuals. By determining the gut microbiota alterations in diabetes, we hope to provide new treatment strategies to modulate the composition and function of gut microbiota to help in glycemic control.

## Figures and Tables

**Table 1:** The baseline data of participants.

Clinical indexes	DM (*n* = 137)	Con (*n* = 179)	*P* value
Female	62	83	
Male	75	96	
Age (year)	53.29	52	
SBP (mmHg)	130.54 ± 2.77	116.71 ± 1.093	<0.001
DBP (mmHg)	81.01 ± 1.74	72.19 ± 0.94	<0.001
eGFR (mL/min/1.73m^2^)	100.78 ± 3.13	100.33 ± 1.40	0.77
Hb (g/L)	133.26 ± 2.68	143.04 ± 2.18	0.002
GHb (%)	8.43 ± 0.312	5.68 ± 0.05	<0.001
Cr (*μ*mol/L)	65.72 ± 3.43	67.41 ± 1.71	0.34
ALB (g/L)	41.86 ± 0.87	47.34 ± 0.36	<0.001
24 h-pro (g)	0.32 ± 0.12	0.079 ± 0.004	0.003
GRAN (×10^9^)	4.04 ± 0.36	3.29 ± 0.12	0.023
LYM (×10^9^)	1.81 ± 0.11	1.80 ± 0.06	0.887
T/Cr (g/g)	32.74 ± 4.24	0.085 ± 0.004	<0.001

SBP: systolic blood pressure; DBP: diastolic blood pressure; eGFR: estimated glomerular filtration rate; Hb: hemoglobin; GHb: glycosylated hemoglobin; Cr: creatinine; ALB: serum albumin; 24 h-pro: 24 h urine protein; GRAN: neutrophil count; LYM: lymphocyte count; T/Cr: taurine to creatinine ratio in random urine.

## Data Availability

The data of this study is available upon request from the corresponding author.
